# Exploring the relationship between loneliness and volunteering amongst Australian adults: a cross-sectional study

**DOI:** 10.1186/s12889-024-17807-4

**Published:** 2024-01-23

**Authors:** Tara Williams, Ali Lakhani, Evelien Spelten

**Affiliations:** 1 Department of Rural Nursing and Midwifery, La Trobe Rural Health School, La Trobe University, PO Box 4095, Mildura, VIC 3500 Australia; 2 School of Psychology and Public Health, La Trobe University, Melbourne, VIC 3086 Australia; 3 La Trobe Rural Health School, La Trobe University, Bendigo, VIC 3552 Australia

**Keywords:** Loneliness, Volunteer, Social connection, Cross-sectional, Australian

## Abstract

**Background:**

Alleviating loneliness and fostering social connections and a sense of belonging are essential for individuals’ well-being in the aftermath of the COVID-19 pandemic. Volunteering has emerged as a potential strategy to prevent or alleviate loneliness among adults. To gain insights into factors that can reduce or protect against loneliness, it is important to consider multi-dimensional measures of loneliness and motivations to volunteer. This study aimed to understand which variables predict Australian volunteers’ social, family, and romantic loneliness.

**Methods:**

From October 2021 to January 2022, a cross-sectional online survey was administered to a sample of Australian adults with volunteering experience. The survey collected demographic information and used validated measures to assess social, family, and romantic loneliness and volunteer motivation. Bivariate and multivariate analyses were conducted to examine the association between loneliness and motivations for volunteering.

**Results:**

Of the 1723 individuals who accessed the survey link, 160 participants completed the survey. The average age of respondents was 59.87 years (SD 12.3). The majority were female (77.5%), married or partnered (70.6%), and had ten or more years of volunteering experience (62.1%). Overall, participants reported low to moderate levels of loneliness across social (M = 12.1; SD = 5.3), family (M = 11.3; SD = 6.7), and romantic (M = 14.8; SD = 8.3) dimensions. Social motivation for volunteering was negatively associated with social and romantic loneliness, while enhancement and protective motivations were positively associated with family and romantic loneliness. Age and rurality were not significantly associated with any dimension of loneliness.

**Conclusions:**

Loneliness is a multifaceted and intricate experience that impacts individuals socially and emotionally. This study’s findings confirmed that having protective and enhancement motives to volunteer was associated with increased loneliness. Social motives to volunteer were associated with a tendency to have lower levels of loneliness, possibly due to the positive impact of building and maintaining social relationships through volunteering. Understanding these associations is necessary to ensure that volunteering activities align with each person’s unique needs and motivations.

## Background

Loneliness is increasingly recognized as a significant global public health issue [1]. The COVID-19 pandemic amplified this growing concern, confirming how significant a person’s social connections are to their health and well-being [2]. Whilst social isolation refers to the structure of a person’s social network or the objective situation of being alone or lacking social relationships [3], loneliness is the subjective, unwelcome feeling that occurs when there is a discrepancy between a person’s actual and desired relationships with others, such as the quality of the relationships or the lack of a particular relationship [4]. Weiss [5] proposed different dimensions of loneliness, known as social loneliness and emotional loneliness. Social loneliness is a deficit in social relations triggered by events such as moving to a new town and is best addressed by approaches such as joining a new social group [5]. In comparison, emotional loneliness is caused by the absence of a specific personal relationship, whether of a loving partner or friends, who provide acceptance and understanding [5, 6]. Recent estimates indicate that loneliness affects approximately one-third of the population in developed countries with one in 12 people experiencing loneliness at problematic levels [7]. In a 2018 survey, over half (50.5%) of Australian adults reported feeling lonely for at least one day a week, while 27.6% reported feeling lonely for three or more days [8]. During the COVID-19 pandemic, loneliness increased to 54% [9], with 28% of Australians experiencing social loneliness and 19.8% feeling emotionally lonely [10]. Problematic levels of loneliness can lead to reduced quality of life and decreased functioning, affecting physical health [11–18] and mental health outcomes [19–23]. Chronic loneliness and social isolation increase the likelihood of premature death [17, 24, 25], with a recent meta-analysis reporting that social isolation was associated with a 32% higher likelihood of all-cause mortality, while loneliness was associated with a 14% increase likelihood of early death [26, 27]. Considering the evidence demonstrating the detrimental impacts social disconnection has on poor health outcomes, finding interventions that alleviate or protect against loneliness and foster social connection is essential.

One approach toward reducing loneliness and providing physical and psychosocial benefits for individuals is through formal volunteering. The *Cacioppo Evolutionary Theory of Loneliness (ETL)* suggests loneliness is a warning signal indicating a lack of social connections that motivates people to resolve negative feelings triggered by an unmet need to belong [7]. Volunteering allows people to connect in social groups and may assist in avoiding or alleviating loneliness. Research suggests many health and social benefits are associated with volunteering, including increased self-rated health and life satisfaction [28], improved quality of life [29–31], enhanced perceived control and self-efficacy [32], self-rated health and psychological well-being [29, 33, 34] and happiness [35]. Whilst much of the evidence focuses on the health and psychosocial benefits, less research is available investigating the relationship between loneliness and volunteering [30–32, 36–38]. The limited available evidence suggests formal volunteering can partially mediate the relationship between loneliness and quality of life [32, 39], alleviate loneliness [40], protect against loneliness [32, 41], and moderate or higher intensity volunteering (more than 100 h per year) can reduce the risk of loneliness [36, 41, 42]. Studies have also researched the association between other and self-orientated volunteering and self-esteem, well-being, self-efficacy, social connectedness and social trust [43], mental and physical health, life satisfaction, social well-being and depression [44] and mortality [45]. In a study of 4085 Australian volunteers, those with other-orientated motives were more likely to report higher levels of well-being than those who volunteered for self-oriented reasons and lower levels of social connectedness were associated with higher protective motivation, suggesting those experiencing loneliness may seek out volunteer activities, to escape their personal troubles [43]. Yeung and colleagues investigating the cumulative effects of other and self-orientated volunteering found that other-oriented volunteering had significantly stronger effects on mental and physical health, life satisfaction, and social well-being outcomes than self-oriented volunteering, with the strongest impact of other-oriented volunteering on social well-being (social integration, social acceptance, and relationship satisfaction) [44].

Whilst loneliness research continues to grow worldwide, there is a recognized lack of published research within the Australian context [8, 46], particularly looking at the association between loneliness and volunteering. In a recent systematic review investigating care giving, loneliness and volunteering, two studies from the USA and one each from the UK, China, Netherlands and New Zealand directly reported on the association between loneliness and volunteering, with none conducted in Australian populations [47]. Understanding the association between loneliness and volunteering in an Australian context is necessary due to the potential benefits of volunteering in mitigating loneliness. The Australian volunteering experience has a unique history, geography, demography, cultural and social dynamics, and volunteer practices [48, 49]. Providing insight into what motivates a person to volunteer and how it is associated with different dimensions of loneliness is essential to developing more effective volunteer interventions designed to reduce loneliness and increase social connection. This study aimed to understand which variables predict social, family, and romantic loneliness in a group of Australian adults who volunteered and addressed through the following research questions:

1. What is the association between loneliness and motivation to volunteer in an Australian context?
2. What variables predict loneliness among individuals engaged in volunteering in Australia?

## Methods

### Study design

This study used a cross-sectional design and analyzed data from an online survey conducted in Victoria, Australia. The researchers used social media recruitment to overcome mandated social distancing and extended lockdowns imposed by public health directives during COVID-19. The questionnaire was developed and hosted on the Question Pro platform [50] and distributed through a specific Facebook page set up for the study. The instruments were designed to measure individual factors related to loneliness (emotional and social), volunteer motivations and participation and demographic variables [51]. The choice of measurement tools was informed by other studies [43, 52]. Ethics approval was obtained from the University Ethics Committee under the approval number HEC21268.

### Recruitment and participants

Adults over 18 years with volunteering experience and living in Victoria, Australia, were eligible to participate in the survey. Victorian residents were targeted through Facebook advertisements limited to Victorian postcodes. During recruitment, participants confirmed residency through postcode data and self-reported their volunteer status and how many years they had volunteered. Recruitment occurred over 15 weeks, from October 2021 to January 2022. Respondents accessed the survey link on mobile devices or desktop computers and completed electronic consent before commencing the survey. Recruitment strategies included boosted posts to promote posts on potential participants’ Facebook feeds and purposive and snowball recruitment through sharing posts on relevant Facebook pages and requests for volunteer organizations to circulate the survey link via Facebook.

### Outcomes and measures

#### Outcome variable

This research includes a more comprehensive measure of the multiple dimensions of loneliness to distinguish between social, family and romantic loneliness. The primary outcome variables were (social) loneliness and emotional loneliness (family and romantic), measured using the Social and Emotional Scale for Adults – Short (SELSA-S − 15- item) [52]. The 15-item Likert scale comprises three subscales (social, family and romantic) of loneliness. Each subscale consists of five statements about feelings of loneliness within the past year. Items were rated on a 7-point Likert-type scale that ranges from 1 (strongly disagree) to 7 (strongly agree). Mean scores are calculated for each subscale, and higher SELSA-S scores indicate higher levels of loneliness in the domain. The social loneliness subscale includes items 2, 3, 4, 9, and 11, measures feelings toward being part of a social group, and contains statements such as: “I can depend on friends for help”. The family loneliness subscale includes items 2, 5, 17, 19, and 23 and assesses feelings toward family relationships, including statements such as “I feel close to my family”. The romantic loneliness subscale is composed of items 4, 8, 10, 15, and 21. It measures the degree to which participants feel they have significant others in their lives and includes statements such as “I have a romantic or marital partner who gives me the support and encouragement I need”. The SELSA-S’s three subscales have high internal reliability, with Cronbach’s alpha coefficients ranging from 0.87 to 0.90, and were found to be a valid measure of loneliness [52].

#### Predictor variable

The *Volunteer Functions Inventory* (VFI) [53] was used to measure six recognized motivational functions of volunteering (values, understanding, enhancement, protective, social and career). The VFI consists of 30 statements on volunteering to which respondents indicate the importance of each one using a 5-point Likert scale, ranging from 1 = *not important* to 5 = *extremely important*. Mean scores for each motivation subscale were calculated, with higher VFI scores indicating higher motivation levels in the functional area. The values subscale includes items 3, 8, 16, 19, and 22, measures volunteers seeking to express prosocial and humanitarian values through action and contains statements: “I feel it is important to help others”. The understanding function includes items 12, 14, 18, 25, and 30. It assesses volunteers seeking to learn more about the world, other people, and their own skills, including statements such as “volunteering lets me learn things through direct, hands-on experience”. The enhancement subscale is associated with looking to feel needed and good about themselves, with a statement such as “volunteering makes me feel needed” and includes items 5, 13, 26, 27, and 29. The protective function contains items 7, 9, 11, 20, and 24, with volunteers seeking to distract themselves from their own problems or to reduce guilt about being more fortunate with a statement such as “by volunteering, I feel less lonely”. The social subscale is measured with items 2, 4, 6, 17, and 23, with volunteers seeking to reinforce bonds with friends and family who volunteer, including statements such as “my friends volunteer”. A career function includes items 1, 10, 15, 21, and 28. It assesses volunteers seeking benefits to assist them with paid employment opportunities, including statements such as “volunteering allows me to explore different career options”. The *VFI’s* six subscales have high internal reliability, with Cronbach’s alpha coefficients ranging from 0.87 to 0.90, and are a valid measure of volunteer motivation [53].

#### Socio-demographics

Demographic data collected included predictors of loneliness identified from previous studies [54], including age, gender, ethnicity, disability status, marital status (married, never married, widowed, separated/divorced), living arrangements (living alone, living with a spouse, living with friends or family), area of residence using the Australian Statistical Geography Standard (ASGS) (major cities, inner regional, outer regional and remote areas) [55] mode of transport, education level (no formal education to university level) employment, and socio-economic status, measured by the Socio-Economic Indexes for Areas (SEIFA) [56].

#### Analysis

Scores for the SELSA-S subscales and VFI subscales were calculated according to the instructions provided by the developer. Data were processed using the Statistical Package for the Social Sciences software package (v28.0, SPSS Inc., Chicago, IL, USA). All categorical variables (gender, marital status, area of residence, mode of transport, living arrangements, and employment) were converted to dummy variables for the regressions. For example, living arrangements were categorized into live alone and live with others (1/0), with people who lived with a partner, family and friends grouped into live with others. The Shapiro-Wilk test revealed the *social*, *family* and *romantic* loneliness variables were not normally distributed; therefore, non-parametric methods, including Spearman rank correlation, Kruskal-Wallis test and Mann -Whitney U test, were used for the bivariate analysis. Descriptive analyses were conducted to determine the sample characteristics, including frequencies and mean with standard deviation (SD). We then undertook bivariate analysis to examine relationships between the dependent variables (*social, family* and *romantic* loneliness) and the volunteer motivation subscales as the independent variables (RQ1). To address RQ2, three multiple regression models were developed, examining the relationships between *social*, *family* and *romantic* loneliness and multiple independent variables statistically correlated with loneliness in the bivariate analysis.

## Results

### Descriptive analysis

#### Participant characteristics

A total of 1723 clicks on the survey link resulted in 237 participants commencing the survey with 160 complete responses (67.93% completion rate). Most respondents were female (77.5%), aged 18–90 years (M = 59.87, SD = 12.3). 53.1% had a university degree, 70.6% were married or partnered, 53.8% reported living with a spouse or partner, 11.3% were divorced, and 11.3% were single. The majority of respondents (41.5%) lived in inner regional areas (31.3%) lived in outer regional and remote areas, and (27.4%) lived in major cities. Results revealed that (42.5%) were retired, (38.8%) were employed either full- or part-time, and the majority (62.1%) had ten or more years of volunteering experience. A total of 82.5% of respondents indicated they drove a car as their primary mode of transport. A summary of the key characteristics of the sample is presented in Table 1.

#### SELSA-S loneliness

The SELSA-S subscales for the current study were reliable and produced Cronbach’s alpha coefficients of *social* (ɑ = 0.77), *family* (ɑ = 0.90), and (ɑ = 0.85) for the *romantic* dimensions. The overall scores for loneliness were small to moderate across all SELSA-S subscales. The means ranged from 12.1 (SD = 5.3) for the *social* loneliness subscale, 11.3 (SD = 6.7) for the *family* loneliness subscale and 14.8 (SD = 8.3) for the *romantic* loneliness subscale, with higher values indicating greater perceived loneliness. For results, see Table 2.

**Table 1:** Descriptive characteristics

Description	N = 160 (percent %)	Missing values (percent %)
**Gender**		n = 1 (0.6)
Female	124 (77.5)	
Male	34 (21.3)	
Non-binary	1 (1.3)	
**Age**		n = 3 (1.9)
18–25	3 (1.9)	
26–35	2 (1.3)	
36–45	17 (10.6)	
46–55	29 (18.1)	
56–65	47 (29.4)	
Over 65	59 (36.9)	
**Descent**		n = 1 (0.6)
Australia	138 (86.3)	
Other	21 (13.9)	
**Area of residence**		n = 1 (0.6)
Major cities	43 (26.9)	
Inner regional	66 (41.3)	
Outer regional and Remote	50 (31.3)	
**Year spent volunteering**		n = 7 (4.4)
1–5	39 (24.4)	
6–9	19 (11.9)	
Ten years or more	95 (59.4)	
**Level of education**		n = 1 (0.6)
Secondary School	37 (23.1)	
Trade/TAFE	37 (23.1)	
University	85 (53.1)	
**Partnership/Marriage**		n = 1 (0.6)
Married or Partnered	113 (70.6)	
Single	21 (13.1)	
Separated/Divorced	18 (11.3)	
Widowed	7 (4.4)	
**Employment**		n = 3 (1.9)
Working	61 (39.4)	
Retired/Pension	74 (46.2)	
Carer	6 (3.8)	
Unemployed	11 (6.8)	
Studying	5 (3.1)	
**Living arrangements**		n = 1 (0.6)
Lives with spouse/partner	86 (53.8)	
Lives alone	37 (23.1)	
Live with family	33 (20.6)	
Lives with friends	3 (1.9)	
**Transport**		n = 1 (0.6)
Car – as driver	132 (82.5)	
Other	27 (16.9)	
**Live with disability**		n = 1 (0.6)
Yes	25 (15.6)	
No	134 (83)	

**Table 2:** Descriptive statistics for the three SELSA-S subscales

	N	Mean	SD	Minimum	Maximum
Social loneliness	160	12.1	5.3	5	28
Family loneliness	160	11.3	6.7	5	35
Romantic loneliness	160	14.8	8.3	5	35

### Bivariate analysis

#### Volunteer motivation and loneliness

In the present study, Cronbach’s alpha coefficients for the *VFI* were as follows: values (ɑ = 0.79), understanding (ɑ = 0.81), enhancement (ɑ = 0.85), protective (ɑ = 0.81), social (ɑ = 0.82), and career (ɑ = 0.90). Significant correlations were associated with social, enhancement and protective motivations and loneliness; however, career, values and understanding motivations did not correlate with any loneliness subscales. There were significant negative correlations between social motivation and social loneliness (rs = -0.28, n = 160, *p* < 0.01) and social motivation and romantic loneliness (rs = -0.16, n = 160, *p* < 0.05. Significant positive correlations were found between family loneliness and enhancement motivation (rs = 0.19, *p* < 0.05) and protective motivation (rs = 0.28, *p* < 0.001) and romantic loneliness and enhancement motivation (rs = 0.17, *p* < 0.05), and protective motivation (rs = 0.19, *p* < 0.05). For results of bivariate analysis between loneliness and volunteer motivation see Table 3.

**Table 3:** Spearman’s rank effect size between social, family and romantic loneliness and volunteer motivation

	Social loneliness	Family loneliness	Romantic loneliness
VFI Career (n = 160)	-0.04	0.11	0.01
VFI Social (n = 160)	-0.28**	-0.03	-0.16*
VFI Values (n = 160)	-0.13	0.03	-0.01
VFI Enhance (n = 160)	0.12	0.19*	0.17*
VFI Protect (n = 160)	0.13	0.28**	0.19*
VFI Understand (n = 160)	-0.07	0.04	0.07

**Correlation is significant at the 0.01 level (2-tailed)

*Correlation is significant at the 0.05 level (2-tailed)

### Sociodemographic variables

Urban areas (major cities) had slightly higher *social*, *family*, and *romantic* loneliness scores than regional and remote areas; however, these were not statistically significant. No significant difference was found between age and the three loneliness subscales. *Social* loneliness scores were significantly higher in males (*Md* = 12.0, IQR = 7.0, n = 34) compared to females (*Md* = 10.0, IQR = 8.00, n = 124), U = 1681.5, z = − 2.04, *p* = 0.05, with a small effect size *r* = 0.16. Significantly higher *social* loneliness scores were revealed amongst those living alone (*Md* = 15.0, IQR = 8.0, n = 36), those living with disability (*Md* = 15.0, IQR = 8.0, n = 25), and those with vocational education (*Md* = 13.0, IQR = 8.5, n = 37). Significantly lower *social* loneliness scores occurred in females (*Md* = 10.0, IQR = 8.0, n = 124), those married or partnered (*Md* = 10.0, IQR = 7.0, n = 113) and those with a university education (*Md* = 10.0, IQR = 7.5, n = 85). Significantly lower *social* loneliness scores were associated with volunteering for ten years or more (*Md* = 10.0, IQR = 7.00, n = 95) in comparison to 6–9 years (*Md* = 11.0, IQR = 8.0, n = 19) and 1–5 years (*Md* = 13.0, IQR = 8.0, n = 39). The findings indicate that living alone (*Md* = 12.0, IQR = 10.5, n = 37) and living with disability (*Md* = 12.0, IQR = 10.5, n = 25) are both associated with increased *family* loneliness, while being married or partnered (*Md* = 9.0, IQR = 7.0, n = 113), driving a car *(Md* = 9.0, IQR = 8.00, n = 132), and being retired (*Md* = 8.0, IQR = 5.8, n = 68) are all significantly associated with reduced *family* loneliness. *Romantic* loneliness scores were significantly higher amongst those living alone (*Md* = 23.0, IQR = 9.5, n = 37) and those who had a vocational education (*Md* = 17.0, IQR = 14.0, n = 37) and significantly lower in those who were married or partnered (*Md* = 9.0, IQR = 8.5, n = 113) and those with a university education (*Md* = 13.0, IQR = 14.0, n = 85), see Table 4.

**Table 4:** Non-parametric tests for significant difference

Variable	Social loneliness		Family loneliness		Romantic loneliness	
	Mann-Whitney U or Kruskal Wallis H statistic	*P*-Value	Mann-Whitney U or Kruskal Wallis H statistic	*P*-Value	Mann-Whitney U or Kruskal Wallis H statistic	*P*-Value
Gender	0.16^U^			0.46		0.60
Age		0.27		0.15		0.28
Area of residence		0.16		0.33		0.19
Volunteer years^b^	9.77^H^			0.44		0.16
Vocational education	0.16^U^			0.38	0.20^U^	
University education	0.20^U^			0.17	0.16^U^	
Married or Partnered	0.24^U^		0.26^U^		0.62^U^	
Retired		0.09	0.26^U^			0.1
Live alone	0.23^U^		0.20^U^		0.55^U^	
Live with disability	0.21^U^		0.28^U^			0.11
Drive a car		0.09	0.26^U^			0.19

*U =* Mann-Whitney U statistic

*H=* Kruskal-Wallis H statistic

### Multivariate analysis

#### Social loneliness

A backward multiple regression was conducted using SPSS software with eight independent variables that correlated significantly with *social* loneliness in the bivariate analysis (gender, married/partnered, living alone, living with disability, volunteer years, vocational education, university education and social motivation), removing independent variables that did not contribute significantly to the model. The factors that best explained *social* loneliness included living with disability, years of volunteering, university education and social motivation, explaining 21 per cent of the variance (*F* = 7.5, adjusted *R2* = 0.2, *p* < 0 0.001). Years spent volunteering (*β*= -0.24, *t*= -3.2, *p =* 0.002), university education (*β*= -0.19, *t*= -2.4, *p* = 0.02), and social motivation (*β*= -0.29, *t*= -3.9, *p* < 0.001) were significantly negatively associated with *social* loneliness and living with disability (*β* = 0.19, *t* = 2.5, *p* = 0.01), was significantly positively associated with *social* loneliness. The results showed that gender and living alone were not significantly associated with social loneliness, see Table 5.

**Table 5:** Output for backward multiple regression analysis prediction of social loneliness

Variables						95% confidence interval	
	B^b^	SE	Beta(β)^c^	t	p	Lower limit	Upper limit
(Constant)	22.6	2		11	< 0.001	19	27
Volunteer years	-1.45	0.5	-0.24	-3.2	0.002	-2.4	-0.6
VFI social	-0.34	0.1	-0.29	-3.9	< 0.001	-0.5	-0.2
Live with disability	2.8	1	0.19	2.5	0.01	0.6	5.0
Female gender	-1.74	1	-0.13	-1.7	0.07	-3.6	0.2
University level	-2.0	0.8	-0.19	-2.4	0.02	-3.6	-0.4

^a^Dependent Variable: SELSA S

^b^Unstandardized Coefficient (*B*)

^c^Standardized Coefficient (β)

### Family loneliness

**Table 6:** Output for backward multiple regression analysis prediction of family loneliness

Variables						95% confidence interval	
	B^b^	SE	Beta(β)^c^	t	p	Lower limit	Upper limit
(Constant)	10.7	1.6		6.8	< 0.001	7.6	14
Married or partnered	-3.52	1	-0.24	-3.3	0.001	-5.6	-1.4
Live with disability	3.07	1.3	0.17	2.3	0.02	0.4	5.7
Protective motivation	0.35	0.1	0.23	3.1	0.002	0.1	0.6
Retired	-3.12	1	-0.23	-3.3	0.001	-5.0	-1.2

^a^Dependent Variable: SELSA F

^b^Unstandardized Coefficient (*B*)

^c^Standardized Coefficient (β)

We ran the second backward multiple regression to determine the combined effect of seven predictor variables correlated significantly with *family* loneliness in the bivariate analysis (mode of transport, retirement, married/partnered, living alone, living with disability, protective and enhancement motivation). The factors that best explained *family* loneliness included being married or partnered, protective motivation, retired, and living with disability, explaining 23 per cent of the variance (*F* = 13.1, adjusted *R2* = 0.23, *p* < 0.001). Being married or partnered (*β*= − 0.24, *t*= -3.3, *p* = 0.001) and being retired (*β*= -0.23, *t*= -3.3, *p* = 0.001) were significantly negatively associated with *family* loneliness and living with disability (*β* = 0.17, *t* = 2.3, *p* < 0.05) and protective motivation (*β* = 0.23, *t* = 3.1, *p* < 0.01) were significantly positively associated with family loneliness, see Table 6.

### Romantic loneliness

The final backward multiple regression was run to determine the linear combination of seven independent variables (married/partnered, living alone, university education, vocational educational, social, protective and enhancement motivation) significantly correlated with *romantic* loneliness in the bivariate analysis. The factors that best explained r*omantic* loneliness were being married or partnered, protective motivation, social motivation and those with vocational education, explaining 48 per cent of the variance (*F* = 37.5, adjusted *R2* = 0.48, *p* < 0.001). Being married or partnered (*β*= − 0.62, *t*= -10.6, *p* < 0.001) and social motivation (*β*= − 0.16, *t*= -2.4, *p* < 0.05) were significantly negatively associated with romantic loneliness, and those with vocational education (*β* = 0.14, *t* = 2.3, *p* < 0.05) and protective motivation (*β* = 0.22, *t* = 3.4, *p* < 0.001) were significantly positively associated with romantic loneliness, see Table 7.

**Table 7:** Output for backward multiple regression analysis prediction of romantic loneliness

Variables						95% confidence interval	
	B^b^	SE	Beta(β)^c^	t	p	Lower limit	Upper limit
(Constant)	20.5	1.7		12	< 0.001	17	24
Married or partnered	-11.3	1.1	-0.62	-10.6	< 0.001	-13	-9.2
Protective motivation	0.42	0.1	0.22	3.4	< 0.001	0.2	0.7
Social motivation	-0.28	0.1	-0.16	-2.4	0.02	.-0.5	-0.1
Vocational education	2.64	1.1	0.14	2.3	0.02	0.4	4.9

^a^Dependent Variable: SELSA R

^b^Unstandardized Coefficient (B)

^c^Standardized Coefficient (β)

## Discussion

### Main findings

In this study, we conducted an online cross-sectional survey to examine the relationship between loneliness and volunteer motivation among Australian adults using a multi-dimensional measure of social, family, and romantic loneliness. Overall, participants reported low to moderate levels of *social family* and *romantic* loneliness. Social motivation, being married or partnered, having a university education, being retired, and having more years of volunteering experience were significant negative predictors of loneliness. On the other hand, protective motivation, living with a disability, and having a vocational education were identified as significant positive predictors of loneliness in the multivariate analysis. The findings will be discussed in further detail under the main headings of volunteering and loneliness, sociodemographics and loneliness and future research.

#### Volunteering and loneliness

This study occurred during the COVID-19 pandemic, which limited people’s ability to leave their homes and significantly disrupted volunteering globally and within Australia. With restrictions on social and physical distancing and imposed lockdowns, many volunteer-involving organizations cancelled or postponed volunteering to protect volunteers’ health and well-being (Mao et al., 2021; Volunteering Victoria, 2020), and some volunteers decided to reduce or stop volunteering altogether [57, 58]. We examined the relationship between loneliness and six volunteer motivations that fulfil a person’s unique needs and motives and which can evolve over time [53, 59]. Our results found that protective and enhancement motivations were positively associated with *family* and *romantic* loneliness. Mayer, Fraccastoro and McNary referred to this combined enhancement/protective function as a “sense of worth” [60], which may motivate individuals to volunteer as a means to alleviate their painful feelings of loneliness and seek validation and acceptance (protective function) [43, 53, 59], whilst the enhancement function may motivate a person to volunteer to boost their self-esteem and feel more important and needed [53]. Furthermore, our findings indicated that social motivation to volunteer was negatively associated with *social* loneliness. In this way, those with social motivates to volunteer may experience lower levels of *social* loneliness due to the positive impact of building and maintaining social relationships through volunteering. Individuals with social motives tend to volunteer to increase their social interactions, establish new friendships, strengthen existing relationships, and gain the approval of others [43, 53, 59]. Volunteering driven by social motivation (other–orientated) has also been linked to improved health outcomes [43, 61], lower rates of depression and mental health issues [44], and reduced mortality [45]. Furthermore, participants who had engaged in volunteer activities for ten years or more had significantly lower *social* loneliness scores compared to those with shorter volunteering periods. Older people engaged in volunteering tend to experience lower levels of loneliness levels than non-volunteers [10, 47], particularly when they volunteer more than 100 h per year [36, 42]. The findings from our study support the idea that for some people, volunteering may act in a way that helps them build and maintain social relationships with others or to feel needed, important and helpful to others and in this way, help protect against or reduce family loneliness [62]. Volunteering can give adults a sense of purpose, belonging, and meaning, as they feel valued and appreciated while giving back to their community [32]. Given the significant impact of social connections on health and well-being, our findings hold significance as they highlight the potential benefit of formal volunteering in supporting individuals experiencing or at risk of loneliness.

### Sociodemographics

#### Marital status and loneliness

One interesting finding from this study is that *family* loneliness scores were the lowest among all dimensions of loneliness. In our study, being married or partnered was a significant negative predictor of *family* loneliness. With 75% of participants reporting living with their spouse/partner or family members, the low *family* loneliness scores may indicate that respondents spent extra time with household members they would not usually be at home with during the day, strengthening those relationships and contributing to lower *family* loneliness. In addition, during times of social distancing, individuals may have made extra efforts through increased use of virtual communication to stay in contact and celebrate with family members to maintain and nurture meaningful connections. A study investigating how Australians maintained connections during COVID-19 lockdowns found that a significant majority (93%) of respondents contacted family members living elsewhere at least once a week using methods such as talking, texting, and innovative approaches like virtual meals, playing games, reading stories, or watching movies to stay connected [63]. Despite the lower levels of *family* loneliness, it is worth mentioning that *romantic* loneliness emerged as the highest of all loneliness subscales. In our findings, although 71% of respondents reported being married or partnered, only 54% lived with their partner/spouse, suggesting that some may have experienced physical separation from their partners, potentially influenced by the COVID-19 lockdowns in Victoria. A study conducted during COVID-19 in Germany found that those with higher relationship satisfaction experienced reduced loneliness, and those with a partner but who lived separately reported loneliness comparable to those who lived alone or were single [64]. Consistent with previous research, romantic relationships have been shown as a protective factor against loneliness and an essential source of social and emotional support [8, 64] and highlighting the association between the absence or loss of a partner and increased loneliness [10, 65]. Decreased romantic relationship satisfaction may have also impacted the higher *romantic* loneliness due to increased relationship strain caused by children’s homeschooling and the complex job demands and economic pressures associated with working from home [66]. For those who reported living alone (23%), lockdowns and restrictions on leaving their home may have increased social isolation and hindered the development of new romantic connections [67], leading to increased *romantic* loneliness. The reasons people live alone or are single are complex; however, it is important to recognize the contribution these factors can have on a person’s social disconnection and emotional loneliness [5, 68]. While volunteering is often viewed as a potential solution to loneliness, it is important to recognize that alleviating romantic loneliness requires a solid and intimate relationship with a partner or close friend [5]. However, volunteering can offer lonely people opportunities to broaden their social networks, build new social connections, and receive additional social support and resources, even if their existing social and family relationships may not entirely alleviate their sense of loneliness.

#### Education level and loneliness

The level of education was also a significant sociodemographic factor in our findings, with those with a university education associated with less *romantic* loneliness and those with a vocational education associated with increased *romantic* loneliness. Research indicates that individuals with a higher level of education are more likely to volunteer [49], consistent with the study’s findings where 53% of participants reported having attained university-level education, whilst (23%) held a vocational level of education. In the context of COVID-19, the lower *romantic* loneliness observed among university-educated individuals may be due to their ability to remain engaged in remote work, allowing them to maintain a sense of normality and a sense of connection with their work colleagues and peers through regular virtual meetings and phone calls [69]. Similarly, for students able to continue their education online, attending classes may have provided a structured routine and regular interaction with their peers and teachers, helping them feel more socially connected and less lonely [70]. The result may have also been influenced by those with a university education having a significant person, such as an intimate partner or family member, also at home who may have provided additional social and emotional support during this challenging time. However, for individuals with vocational education who could not leave home to work, the prolonged lockdowns due to COVID-19 forced people to spend more time together, with less support available from family and friends [66], coupled with employment and economic pressures [71], may have contributed to increased relationship strain, lower relationship satisfaction and increased romantic loneliness. This finding emphasizes the significance of distinguishing between different dimensions of loneliness, highlighting the subjective nature of loneliness and how individuals experience it.

#### Living with disability and loneliness

The study also revealed that living with a disability was a significant positive predictor of *social* and *family* loneliness. The findings of our study may have been influenced by the COVID-19 pandemic, particularly as individuals with disabilities faced greater loneliness due to increased social isolation and the impact of public health restrictions limiting their ability to leave their homes to interact or visit their families. Research has shown that people with disability report higher rates of loneliness compared to non-disabled people peers [72, 73], with 36% of Australians with disability experiencing social loneliness compared to 28% of the general population and 33% report emotional loneliness (defined as a lack of a significant person to whom they have an attachment), compared to 20% in the general population [10]. Individuals with disabilities also experience inequitable access to volunteering [74], which is a significant factor in the context of our findings regarding the association between disability and increased loneliness. This knowledge is particularly relevant when considering strategies to prevent or reduce loneliness through volunteering. It is essential for communities and volunteering organizations to not only effectively engage people with disabilities but also maintain their involvement in inclusive and equitable ways that accommodate changing life circumstances, circumstances, ill health or disability [74, 75]. Providing flexible options, including remote, virtual or micro-volunteering, [49] may help people facing in-person volunteering, including those with disabilities at risk or experiencing loneliness, maintain vital social connections.

#### Future research

The COVID-19 pandemic directly impacted the roles and routines of volunteers, increasing social disconnection, fewer opportunities for social interaction and community engagement and a reduced commitment to volunteering. Future longitudinal research studies are needed to offer a more comprehensive understanding of the longitudinal effects of volunteering in protecting against or reducing loneliness among adult volunteers. Volunteering provides a practical way to bring people together, increase community participation, and improve overall social connection and well-being. It is necessary to gain a deeper understanding of how the experience of volunteering contributes to strengthening social connections and reducing the underlying factors that contribute to loneliness. Recognizing that loneliness is a social issue highlights the importance of finding effective interventions. Social Prescribing (SP) acknowledges the influence of social factors on health and is receiving increased interest globally and within Australia as one approach to address chronic loneliness [76–79]. However, the use of SP as an intervention to address loneliness is still evolving, with a recent systematic review highlighting the need for further research to examine the impact of SP programs on loneliness, isolation, and connectedness [80, 81]. Given the diverse reasons people choose to volunteer, further research is necessary to understand how volunteering strengthens social connections and reduces the underlying factors contributing to loneliness.

#### Strengths and limitations

Our research has notable strengths, which includes using a validated multi-dimensional measure of social, family and romantic loneliness [52], providing a deeper understanding of the complex nature of loneliness compared to using a single measure. Our study gained insights into how geographical location might impact loneliness among volunteers by recruiting participants from a diverse geographical area in Victoria, categorized as major cities, inner regional, outer regional and remote areas based on their postcodes, which previous studies have often neglected [82]. However, it is important to acknowledge the limitations of our research. Firstly, fewer people volunteering within the target population during the COVID-19 pandemic may have impacted recruitment and the small sample size [48]. The power analysis provided an estimated sample size; however, the findings should be interpreted with caution, considering the assumptions made and the dynamic nature of the research context [83]. Participants were recruited through Facebook to an online survey, which may have restricted our sample to those with internet access, digital literacy and proficiency in English. It is important to also recognize the limitations of self-reported data on the accuracy of volunteer status and postcode data used for participant recruitment, which may have impacted the accuracy of results. There was also an underrepresentation of minority groups, such as First Nations Peoples and those from diverse cultural and ethnic backgrounds, limiting the generalizability of our findings.

## Conclusions

Our study provides insight into the complex relationship between volunteering, loneliness and sociodemographic factors in Australia. Overall, the levels of social, family, and romantic loneliness were found to be low to moderate among people who engage in volunteering. We found that individuals with higher social motivation to volunteer tended to report lower social and romantic loneliness; however, higher enhancement and protective motives for volunteering were associated with higher family and romantic loneliness. Our findings suggest that some people may volunteer to avoid negative feelings or personal troubles and to feel better about themselves whilst others volunteer to strengthen their social relationships with others. Understanding these associations is important for developing targeted interventions to alleviate loneliness and enhance social connection. Loneliness is not one-dimensional, and therefore, while participating in social activities through volunteering can help address social loneliness, it may not fully address the unmet need for close family and intimate relationships associated with family and romantic loneliness. Given the diverse reasons that motivate people to volunteer, it is important to ensure that volunteering opportunities align with each person’s unique needs and motivations.

## Data Availability

The datasets generated and analyzed during the current study are available via figshare at 10.6084/m9.figshare.23293184.

## Abbreviations

SELSA-S: Social and Emotional Scale for Adults – Short
VFI: Volunteer Functions Inventory
SR: Spearman’s’ rank effect size
MW: Mann-Whitney U effect size
SP: Social Prescribing
